# A familial concurrence of schizophrenia and Gaucher's disease

**DOI:** 10.1186/1744-859X-6-33

**Published:** 2007-12-17

**Authors:** Odysseas D Mouzas, Konstantinos E Siomos, Nikiforos V Angelopoulos

**Affiliations:** 1 Department of Psychiatry, Medical School, University of Thessaly, Greece

## Abstract

**Background:**

Gaucher's disease (GD) is the most frequently encountered lysosomal storage disease. Here, we describe and discuss the observed concurrence of schizophrenia and Gaucher's disease in two siblings.

**Methods:**

Presentation of a family with two siblings with Gaucher's disease.

**Results:**

In a six-member family, the first son suffers from schizophrenia, while the third and fourth sons suffer from the Gaucher's disease (type 1 non-neuronopathic). The parents and the second son do not suffer from either illness.

**Conclusion:**

The concurrence of schizophrenia and Gaucher's disease in the same family is an unusual phenomenon. The literature regarding this coincidence is limited, despite the fact that patients with Gaucher's disease have one or two mutated alleles, considered to be a risk factor leading to conditions such as Dementia, Parkinson's disease and schizophrenia.

## Introduction

Gaucher's disease (GD) is the most frequently encountered lysosomal storage disease [1] caused by autosomal recessive inborn defects in the glucocerebrosidase gene (GBA) at the 1q21 chromosome [2,3]. These defects eventually lead to accumulation of the glycolipid in lysosomes within macrophages of the reticuloendothelial system. Clinically, GD can present with a vast phenotypic heterogeneity [4], which can be predicted to some extent from the underlying mutation [5]. Three clinical forms of GD have been identified: type 1 non-neuronopathic, type 2 acute neuronopathic, and type 3 subacute neuronopathic [6]. The concurrence of Gaucher's disease and schizophrenia among siblings is a very rare phenomenon. In the present work, we describe a family with four sons, two of whom suffer from Gaucher's disease and another from schizophrenia, with one being free from any illness.

## Presentation of the family

In this six-member family, the first son displayed paranoid type of schizophrenia and the third and fourth sons displayed Gaucher's disease type 1, non-neuronopathic.

The 23-year-old first son's symptoms, manifested from January 2003 for a period of 5 months. He reported a loss of interest for work, depression of moderate intensity and social withdrawal. In June 2003, he presented with an acute psychotic episode with delusional ideas of control of affect and auditory hallucinations in the form of voices in the third person, depreciatory for the patient. He also had interruptions in the flow of thought, with speech inconsistency, psychomotor excitation and aggressive behaviour directed towards members of his family. No physical illness or illicit substance use were detected. The brain neuroimaging examinations (CT and MRI) were normal. During his psychiatric hospitalisation, he was put on 8 mg risperidone and 4 mg biperiden per day. Psychomotor excitation and aggressive behaviour were ameliorated. However, the delusional ideas and the auditory hallucinations, as well as the disordered thinking, remained. On September 2003, he displayed extrapyramidal symptoms and unexpectedly severe symptoms resembling to tardive dyskinesia. His medication was changed to olanzapine, 10 mg daily, which contributed to the considerable amelioration of these side effects. He was negative for Gaucher's disease on examination.

The 12-year-old brother had been hospitalised for unspecified feverish disease lasting for weeks when he was 9 (2 years before displaying Gaucher's disease), and there were frequent infections in his clinical background. During the clinical and laboratorial examination, cytopenia and splenomegaly were diagnosed. A bone marrow examination showed Gaucher's disease. More detailed tests and general neurological, laboratorial and clinical examinations showed no neurological dysfunction. The final diagnose was Gaucher's disease, type 1, non-neuronopathic. Since that time, he has been under medication with intravenous infusions of glucocerebrosidase. The spleen size has become normal and the cytopenia improved. No other physical or psychiatric disease has been found.

After the diagnosis of Gaucher's disease was made for the patient, the whole family was submitted to special tests for this disease. The control test was negative for the whole family, except the patient's 17-year-old brother. The bone marrow examination for the Gaucher's disease was positive in this case, while inflation in both sides of the knees was observed on clinical examination. There was no neurological involvement, not any other physical or psychiatric disease. Since that time, he has undergone medical treatment with infusion of glucocerebrosidase intravenously each week. No other member of the family suffers from Gaucher's disease or schizophrenia. In fact, the psychiatric examination did not reveal any other psychiatric illness for any other member of the family.

The medical examination did not reveal any other morbidity in any member of the family.

## Discussion

Perez-Calvo et al [7] presented the results of a co-morbidity study regarding Gaucher's disease from a nationwide enquiry in Spain. The aim of that study was to determine if Gaucher's disease patients and non-affected carriers have a risk of suffering from other diseases when compared to healthy non-carrier relatives. The results indicated that the Gaucher's patients or carriers have a relatively higher risk of suffering from any other disease: patient vs healthy 9.69, patient vs carrier 3.74, carrier vs healthy 2.59. The relative risk of suffering from any disease, regarding the patients' and carriers' sex, was 3.96 for female patients and 1.34 for female carriers. This study shows that Gaucher's patients, as a group, seem to have a greater risk, estimated by p values, of suffering from other common unrelated diseases, including schizophrenia, than carriers or healthy relatives. This excess of risk is particularly high among female patients.

Epidemiological studies [8] have demonstrated at least 50% higher rates of schizophrenia among European Ashkenazi Jews, compared to their non-European Jewish counterparts. Type 1 Gaucher's disease is the most common genetic illness among the Ashkenazim. The incidence is estimated to be between 1 in 600 and 1 in 2 500, with a heterozygote frequency of about 1 in 13 in this population [9]. Gaucher's disease is caused by allelic variations of the lysosomal enzyme glucocerebrosidase. The gene locus is at the chromosome 1q21. In particular, the N3705 mutation at the GBA locus on human chromosome 1q21 has a more than 70% frequency in the Ashkenazim [10], and is the second most widespread Gaucher's disease mutation in the European non-Jewish population, with a high frequency among Portuguese and Spanish patients [11,12].

Goodman [13], in order to limit the genetic heterogeneity of schizophrenia, focused on testing the hypothesis that an increased prevalence of the lysosomal enzyme disorders of Gaucher's disease might contribute to the demonstrated increased vulnerability to schizophrenia among the Ashkenazim. The study showed that, among the Ashkenazim, schizophrenic symptoms might be variable phenotypic expressions of the underlying genetic diseases, such as Gaucher's disease.

Herrlin et al [14] reported behaviour disorders and psychotic symptoms in individuals suffering from the juvenile form of Gaucher's disease. Neil et al [15] described a family in which classical non-neuronopathic Gaucher's disease type 1 was years later followed by dementing neurologic and atypical psychotic disorders. The occurrence of Gaucher's disease in two successive generations is extremely rare, and has only been described in three cases. The proband, a 41-year-old woman who had received a diagnosis of asymptomatic Gaucher's disease at the age of 21, had her first psychotic episode at age 40 and a second one a year later. Her clinical presentation was marked by prominent affective lability, depression with intermittent episodes of euphoria, paranoia, multiple phobias, somatic delusions and recurrent impulsive suicidal acts. The proband's 40-year-old brother had been hospitalized on three occasions between the ages of 28 and 32. In addition, he had a medical background with psychiatric admissions to three other hospitals each time after an acute psychotic episode. Gaucher's disease had first been diagnosed by bone marrow examination, 8 years prior to the onset of psychiatric symptoms. The proband's mother, at age 50, displayed paranoid ideation, somatic delusions and movement disorders. A second episode a year later was similar, but more severe. She died at the age of 62 during a complicated surgical procedure on an unexplained fracture on her left hip. Unfortunately, autopsy studies were performed in a perfunctory fashion and examinations that might have demonstrated evidence of Gaucher's disease were omitted.

## Conclusion

The concurrence of schizophrenia and Gaucher's disease in the same family is an unusual phenomenon. The literature regarding the coincidence of Gaucher's disease and schizophrenia in the same family is limited, despite the fact that patients with Gaucher's disease have one or two mutated alleles, considered to be risk factors leading to conditions, such as Dementia, Parkinson's disease and schizophrenia.

## Authors' contributions

ODM made substantial contributions to conception and design of the present study, and in the interpretation of the data. He was also involved in drafting the manuscript and revising it critically. KES made substantial contributions to conception and design of the present study, and in the acquisition, analysis and interpretation of the data. NVA made substantial contributions to conception and design of the present study and in revising critically the manuscript. All authors read and approved the final manuscript.
